# Effectiveness of Acceptance and Commitment Therapy in Central Pain Sensitization Syndromes: A Systematic Review

**DOI:** 10.3390/jcm10122706

**Published:** 2021-06-19

**Authors:** Carmen M. Galvez-Sánchez, Casandra I. Montoro, María Moreno-Padilla, Gustavo A. Reyes del Paso, Pablo de la Coba

**Affiliations:** Department of Psychology, University of Jaén, 23071 Jaén, Spain; imontoro@ujaen.es (C.I.M.); mmpadill@ujaen.es (M.M.-P.); greyes@ujaen.es (G.A.R.d.P.); pcoba@ujaen.es (P.d.l.C.)

**Keywords:** fibromyalgia syndrome, irritable bowel syndrome, chronic tension headache, migraine, interstitial cystitis, temporomandibular disorder, acceptance and commitment therapy, ACT, systematic review

## Abstract

Objectives: Acceptance and commitment therapy (ACT) is considered by the American Psychological Association as an evidence-based treatment for a variety of disorders, including chronic pain. The main objective of the present systematic review was to determine the effectiveness of ACT in patients with central pain sensitization syndromes (CPSS). Methods: This systematic review was conducted according to the guidelines of the Cochrane Collaboration and PRISMA statements. The protocol was registered in advance in the Prospective Register of Systematic Reviews (PROSPERO) international database. The selected articles were evaluated using the Cochrane risk of bias (ROB) assessment tool. The PubMed, Scopus, and Web of Science databases were searched. Results: The literature search identified 21 studies (including investigations of fibromyalgia syndrome, irritable bowel syndrome, and migraine) eligible for the systematic review. There were no studies regarding the effectiveness of ACT for chronic tension-type headache (CTTH), interstitial cystitis (IC), or temporomandibular disorder (TMD). The evaluation of ROB showed that 12 of the selected studies were of low quality, 5 were of moderate quality, and 4 were high quality. ACT reduces some clinical symptoms, such as anxiety, depression, and pain. This positive effect of ACT might be mediated by pain acceptance, psychological flexibility, optimism, self-efficacy, or adherence to values. ACT showed better results in comparison to non-intervention (e.g., “waiting list”) conditions, as well as pharmacological and psychoeducational interventions. It is not entirely clear whether extended ACT treatments are more advantageous than briefer interventions. Conclusions: There are few studies about the effectiveness of ACT on CPSS. However, ACT seems to reduce subjective CPSS symptoms and improve the health-related quality of life of these patients. The absence of studies on the effectiveness of ACT in CTTH, IC, and TMD, indicate the pressing need for further ACT studies in these CPSS.

## 1. Introduction

Central pain sensitization (CPS) results from neuronal plasticity that involves structural and functional changes in the central nervous system (CNS). These changes generate a sustained state of hyperexcitability and excessive synaptic efficiency in the CNS neurons involved in sensory and nociceptive processing [1,2]. CPS can occur both at the brain [3,4] and spinal cord level [5]. In the latter case, CPS promotes activity in ascending modulatory pain pathways [6] and/or dysfunction of descending inhibitory pathways [7]. CPS explains the occurrence of spontaneous pain, and maintenance thereof, as well as hypersensitivity to innocuous (allodynia) or low-intensity (hyperalgesia) stimulation in the so-called central pain sensitization syndromes (CPSS) [2,8,9]. According to the classification developed by M. B. Yunus on central sensitization syndromes (2007; 2009; 2015), those that involve the experience of chronic pain could be considered as CPSS [9,10,11]. In this way, the CPSS under study were: fibromyalgia syndrome (FMS), irritable bowel syndrome (IBS), chronic tension-type headache (CTTH), migraine, interstitial cystitis (IC), and temporomandibular disorder (TMD). In the following lines, a brief explanation of each CPSS will be provided in order to facilitate the understanding of the review.

FMS is characterized by widespread, diffuse, and persistent pain [12]. FMS patients usually present with a wide range of symptoms such as fatigue, sleep disturbances, emotional and affective disorders, cognitive impairments, etc. [13,14,15]. The American College of Rheumatology (ACR) established its diagnostic criteria in 1990, which consisted of (a) widespread pain for at least three months; and (b) pain in 11 of 18 tender points at a pressure of 4 kg [12]. Twenty years later, the ACR modified these criteria, establishing three conditions that must be fulfilled for an FMS diagnosis: (I) meeting the cut-offs for widespread and severe pain on two scales; (II) symptom duration of at least three months; and (III) absence of alternative explanations for the pain [15,16]. Although the prevalence rates of FMS are similar using both sets of diagnostic criteria (slightly higher than 2% of the general population) [17,18], use of the updated ACR diagnostic criteria may increase the prevalence rates [19,20]. In general, a reasonable estimation of FMS prevalence appears to be between 0.5% and 5% in industrialized countries [21].

Regarding the etiopathophysiology of FMS, while its etiology remains unknown, the pathophysiology of this chronic condition seems to be related to the sensitization of CNS processes, e.g., [5,22,23], which underlie the alterations in pain perception displayed by these patients, (e.g., allodynia and hyperalgesia) [24], deficient pain inhibition [25], temporal summation of pain [26] and other indicators of pain sensitization [25,26,27]. Furthermore, dysregulation of both the hypothalamic-pituitary-adrenocortical axis [28,29] and autonomic nervous system [30,31] seem to be also involved in its pathophysiology. Although it is not clear how these physiologic alterations are generated, there is a wide agreement that FMS should be considered as a CPSS [9,10,11,32,33,34].

IBS, a chronic condition affecting the intestine, has signs and symptoms like abdominal pain, bloating, cramping, gas, and changes in bowel movements (as seen in IBS with constipation, diarrhea, or both). The updated Rome diagnostic criteria (ROME-IV) are used for the diagnosis of IBS [35]. The worldwide prevalence of this disorder is difficult to estimate due to the large heterogeneity of the available epidemiological studies; it could be anywhere between 1% and 40%, or even higher, depending on the country. IBS tends to be most prevalent in adults and adolescents, with an estimated rate of 10–20%. Typical of CPSS, IBS is more often in females [36].

Although the etiopathophysiology of IBS remains unknown, several non-mutually exclusive hypotheses have been proposed, including dysregulation of gut motility, visceral hypersensitivity, inflammatory processes, post-infectious processes, microbiomes, food sensitivity, genetics, psychosocial dysfunction, etc. [37]. In addition, disturbances in the spinal modulation of nociception have been reported in these patients [38,39]. Against this background, the similarity in widespread hypersensitivity between IBS patients and CPCS, such as FMS, led to the view that IBS is another CCS [10,11]. This perspective is also coherent with the observed associations between IBS and FMS [9].

IC, also called bladder pain syndrome, is a type of chronic pelvic pain characterized mainly by pain in the bladder, but also in vulvar, suprapubic, pubic, and vaginal areas, along with high urinary frequency, incontinence problems, and nocturia. As well as its unknown etiology, no official criteria for IC diagnosis are available [40]. IC often accompanies other CCS (e.g., FMS and chronic fatigue [41,42] and diseases related to pelvic pain [43,44]. The lack of consistent diagnostic criteria, together with the different comorbidities of IC, lead to underestimation of its prevalence, and wide variability in its reported incidence. The prevalence of IC has been estimated at around 0.5% (according to the O’Leary-Sant survey), but much higher prevalence (>10%) has been found using other diagnostic instruments [45,46].

The pathophysiology of IC seems to be related to dysfunction originating in and around the bladder, adjacent pelvic organs, and the neural tissue in this region. However, the etiologic hypotheses to explain these dysfunctions are unproven or discredited [40]. Some of the most notable etiopathogenic hypotheses are related to epithelial alterations, e.g., [47] or the mentioned central sensitization processes [9]. Regarding the involvement of CS, evidence of central pain amplification has been found in IC; for example, segmental hyperalgesia in response to mechanical pressure stimulation in the suprapubic area (T10–T12) has been observed in IC patients [48]. Accordingly, and due also to the similarities of the symptoms with CSS, IC is also considered a CSS [9,49].

TMD is an umbrella term for various clinical problems in the masticatory muscle complex, temporomandibular joint and associated structures [50]. Signs and symptoms of TMD include pain, impaired jaw function, malocclusion, deviation from the midline on opening or closing of the jaw, limited range of motion, and joint noises and locking [51], together with symptoms such as headaches and sleep disturbances [52]. Regarding TMD prevalence, it is most common in people aged 20–40 years [53]. It has been estimated that approximately 33% of the population have at least one TMD symptom, and 3.6–7.0% of the population have TMD of sufficient severity to necessitate treatment [53]. TMD is one of the most common disorders affecting the maxillofacial region [50].

There is no consensus with respect to the causes, etiological factors, pathophysiology, or management of TMD. In fact, TMD pain continues to be an enigma, and poses a diagnostic and management challenge for many clinicians [54]. However, there is evidence of the involvement of peripheral and CS mechanisms in TMD. Research has focused on the role of the nociceptive system in patients with TMD. Researchers have also assessed trigeminal and extra-trigeminal pain sensitivity in this population. Trigeminal hypersensitivity may be considered to reflect sensitization in the trigeminal area (peripheral sensitization), while extra-trigeminal hypersensitivity is a manifestation of sensitization in distant pain-free areas (CS). In general, there is clear evidence showing that both sensitization processes are involved in the pathophysiology of TMD [55].

CTTH is considered the most prevalent primary headache disorder worldwide [56]. Based on the International Classification of Headache Disorders, third edition (beta version) [57], CTTH is defined by the occurrence of tension-type headache (TTH) on ≥15 days per month, typically with a bilateral, pressing, or tightening quality, mild-to-moderate intensity, and duration of a few hours to days (or unremitting). The pain does not worsen with routine physical activity but may be associated with mild nausea, photophobia, or phonophobia. Due to the exact mechanism of TTH still not being fully understood, use of the term tension-type has been maintained from ICHDI (1988) to ICHD-3 beta [57,58]. Given that there are many similarities and differences between CTTH and chronic migraine (CM), the diagnostic criteria of CTTH have to be improved to allow differential diagnosis between the two disorders [59]. The worldwide prevalence of CTTH is around 0.5–4.8% [59], and it is more prevalent in women [60,61]. Usually, symptoms onset before the age of 10 years; moreover, prevalence seems to decline with age [62].

The etiology of CTTH is not clear. Some studies have pointed out that, in some cases, there is a family history of some form of headache [63] although another study found no significant difference between identical and non-identical twins in CTTH incidence [64]. In general, the mechanisms of CTTH are considered multifactorial, including both peripheral and central mechanisms, as well as genetic and psychological factors. One of the most well-accepted hypotheses states that peripheral pain mechanisms are likely to play a role in episodic TTH, while central mechanisms such as CS might be predominant in CTTH [59].

CTTH may provoke anxiety and interfere with daily life. If CTTH is not treated appropriately, it may worsen symptoms (e.g., analgesia and overuse headache). As a result, effective management is necessary to prevent further complications and improve functionality [65].

Migraine can be conceptualized as a chronic neurological disorder characterized by attacks of moderate to severe headache and reversible neurological and systemic symptoms [66]. The most frequent symptoms are photophobia, phonophobia, cutaneous allodynia, and gastrointestinal symptoms such as nausea and emesis [66]. Moreover, patients with migraine usually report other symptoms such as vertigo, dizziness, tinnitus, and cognitive impairment [67]. The high number and variety of migraine symptoms reflect its complex pathophysiology, and the involvement of multiple neural networks and anatomical regions in the brain [67]. The duration of a migraine headache usually ranges from 4 to 72 h in adults and 2 to 48 h in children. The median time to peak intensity is around 1 h and the median duration is 24 h. Though usually unilateral, pain may be present in any part of the head and frequently occurs in the posterior cervical and trapezius regions [68]. Around a third of people with migraine report reversible neurological symptoms (migraine aura) before the onset, during, and/or in the absence of pain. Migraine with aura is characterized by visual, sensory, language, or disturbances associated with brainstem dysfunction that generally last between 5 and 60 min and occur before the headache [66]. Migraine is recognized as one of the most prevalent and disabling medical illnesses worldwide. The World Health Organization (WHO) ranks migraine as the third most prevalent medical condition and second most disabling neurological disorder in the world [69,70].

The headache phase of migraine is provoked by activation of trigeminal sensory pathways that innervate pain-sensitive intracranial structures, including the eye, dura mater, large cerebral and pial blood vessels, and dural venous sinuses [71]. In individuals with CM, central pain sensitization occurs between the full-blown attacks and could explain the low-grade headache, allodynia, and other symptoms that are characteristic of this disorder [72]. Central sensitization, along with dysfunctional descending pain modulation, could promote the progression and persistence of symptoms, as well as the development of a chronic form of the disease [73].

Acceptance and commitment therapy (ACT; pronounced as a single word, “act”, not as the initials “A-C-T”) is one of the most well-established third-wave therapies [74]. This therapy is based on relational frame theory [75]. ACT states that psychological inflexibility underlies the psychological and emotional suffering, being the main goal of ACT increasing psychological flexibility, defined as the ability to contact the present moment more fully, “as it is and not as what it says it is”, changing or persisting in behavior according the chosen values. Psychological flexibility is based on six core ACT processes (hexaflex model): *acceptance*, active and aware embrace of private events such as thoughts, memories, emotions, and bodily sensations, without unnecessary attempts to change their frequency or form; *cognitive defusion*, attempt to alter the undesirable functions of private events, changing the way one interacts with or relates to them, rather than trying to alter their form, frequency or situational sensitivity; *being present*, contact with private events as they occur using language more as a tool to note and describe the experiences, not so much to predict and judge them; *noticing self*, being aware of experience in relation to the context without attachment to it or to invest in which particular experiences occur; *values*, values are purposively chosen qualities that cannot be obtained, but can be implemented in each moment of everyday life, so not being ends in themselves, rather ways to experience a fuller life; *committed action*, development of a progressively more effective actions linked to chosen values, establishing short, medium, and long-term behavior change goals according to them. Thus, the “suffering” would be based on the opposite processes: *experiential avoidance*, efforts to alter the frequency or form of private events; *cognitive fusion*, excessive literality of language even when it is harmful; *rigidity to the past and future*, attention rigidly toward the past and future, relegating to the “now”; *self as content*, domination of “conceptualized self” over “self as context”; *lack of contact with values*, absence of well-defined and chosen values; *inaction*, inability to change behavior according to the practice of values [74,76].

Some of these processes, such as experiential avoidance, cognitive fusion, or self-as-content seem to be associated with the development and maintenance of psychopathologies and the psychological alterations both in normative [77,78] and clinical or chronic pain populations [79,80,81,82]. Examples of the negative mediator role of these processes are: cognitive fusion mediates the effects of passive coping on anxiety, depression and well-being [78]; or experiential avoidance mediated the effects of rational and emotional copings on depression and stress [79]. Besides, experiential avoidance can have a moderator effect on pain perception [83]. Therefore, part of the chronic pain improvements after ACT interventions would be due to changes in these mediating processes, both in the short-term (pre/post-clinical changes) [84] and long-term (maintenance of these changes after follow-up) [85].

Furthermore, although cognitive-behavior therapy (CBT, which focuses its interventions on the change of the content of experience) has amply demonstrated its efficacy in the treatment of chronic pain patients, the processes explaining its efficacy were not clear. In fact, the main process in which CBT bases its effectiveness, the “cognitive change”, has not been specifically defined and measured in most studies [86]. In this context, ACT provide a consistent theorical model based on altering the ways in which experience influence on the behavior [86].

The American Psychological Association (APA) considers ACT as an evidence-based treatment [87]. Additionally, available evidence points to the neurophysiological brain correlates of clinical improvement after ACT in chronic pain [88,89], supporting the usefulness of ACT in chronic pain populations such as CPSS.

The main objective of the present systematic review was to determine the effectiveness of ACT in the treatment of CPSS. To the best of our knowledge, this is the first systematic review analyzing the effect of ACT on clinical measures of CPSS.

## 2. Materials and Methods

### 2.1. Search Strategy

This systematic review was conducted according to the guidelines of the Cochrane Collaboration and reported according to the Preferred Reporting Items for Systematic Reviews and Meta-Analyses (PRISMA) [90]. The inclusion criteria and analyses were specified in advance, and the protocol was registered in the Prospective Register of Systematic Reviews (PROSPERO) international database (registration ID: CRD42020218208). The search terms were as follows: fibromyalgia syndrome, irritable bowel syndrome, chronic tension headache, migraine, interstitial cystitis, temporomandibular disorder, acceptance and commitment therapy, and ACT.

**Figure 1:**
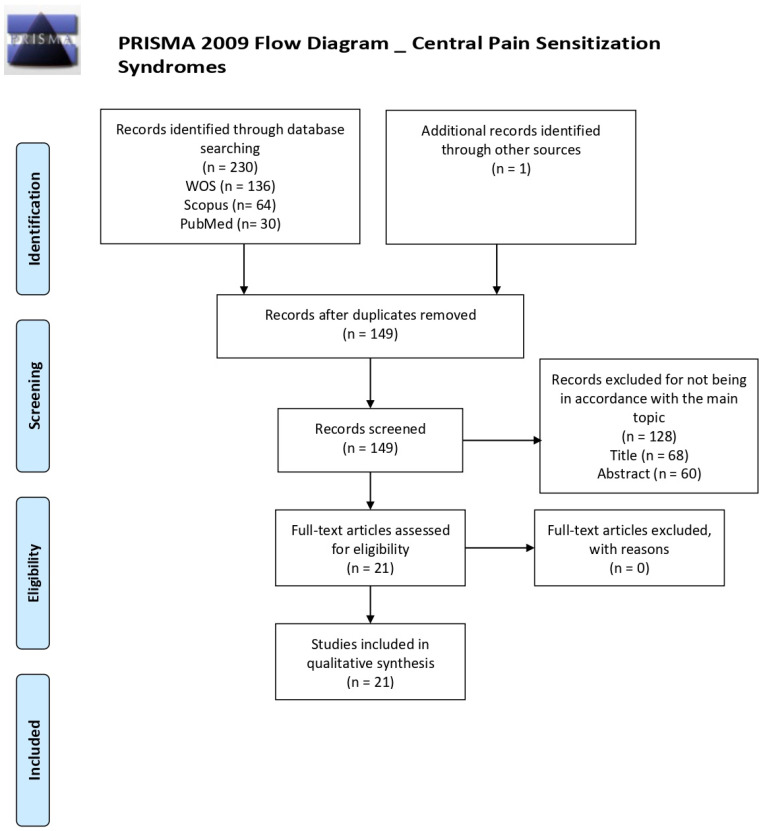
Flow diagram of Central pain sensitization syndromes.

The PubMed, Scopus, and Web of Science databases were searched independently by two researchers. Discrepancies were resolved by consensus. Two reviewers (C.M.G.-S. and P.d.l.C.) independently screened all articles and selected those that satisfied the inclusion criteria for full-text analysis. The titles and abstracts of the articles were screened to remove irrelevant studies; the remaining shortlisted articles were screened in-depth for eligibility. The full-texts of relevant articles were retrieved and screened based on the inclusion and exclusion criteria, to compile a final set of articles to be reviewed. Both reviewers decided whether to include or exclude the articles and any discrepancies were reviewed by the senior author (G.A.R.d.P.), who made the final judgement regarding the inclusion of a study. The screening and selection for inclusion processes are shown as a PRISMA flowchart (Figure 1). Before data extraction and quality assessment, C.I.M. screened all articles in order to confirm their eligibility for this study. The search was restricted to articles published in the past 10 years (the last search was conducted on 1st January 2021).

### 2.2. Eligibility Criteria

Studies were included if they (1) were peer-reviewed original studies of CPSS (including longitudinal studies, pilot studies, pilot randomized controlled trials, randomized controlled clinical, quasi-experimental replicated single-case/small group designs, and uncontrolled and controlled pre/post-test studies), (2) included adult patients (≥18 years old) with CPSS diagnosed using official criteria; and (3) were in English. The exclusion criteria were as follows: (1) review article or meta-analysis; (2) comment, editorial, case report, letter, or meeting/congress abstract; (3) non-English publication; and (4) not a quantitative study.

### 2.3. Data Extraction and Quality Assessment

The study characteristics, methodologies and results were extracted independently by C.M.G.-S. and P.d.l.C., and any discrepancies between them were reviewed by G.A.R.d.P. Data were extracted in the following sequence: first author, study name, country, year of publication, study design, sample size and number of participants in each study group, participant age and sex, and the technique used for CPSS diagnosis. The study characteristics are shown in Table 1. The data were reviewed by G.A.R.d.P. to ensure accuracy of the extraction thereof.

In order to evaluate the quality of the selected articles, both C.M.G.-S. and P.d.l.C. independently evaluated the risk of bias (ROB) in each study according to the Cochrane ROB assessment tool. This tool contains seven items evaluating ROB: random sequence generation (selection bias), allocation concealment (selection bias), blinding of participants and personnel (performance bias), blinding of outcome assessment (detection bias), incomplete outcome data (attrition bias), selective reporting (reporting bias), and other bias. For each item, the ROB was graded as high, medium or low. Discrepancies were resolved by further discussion with the third author. Any discrepancies in the ROB were reviewed by the senior author (G.A.R.d.P.), who made the final decision.

### 2.4. Data Synthesis

In line with our aims, we checked whether the authors compared an ACT group with a non-intervention group (e.g., a “waiting list” [WL] group) or one or more control groups (e.g., pharmacological treatment, psycho-education or alternative therapies groups), or whether there was no comparison group (uncontrolled studies). We also checked whether they adequately reported the results of all groups; studies that calculated effect sizes are detailed in Table 1. Finally, we evaluated the biases of each study and reported these in the Risk of bias section and Table 2.

## 3. Results

### 3.1. Literature Search and Study Characteristics

From among a total of 230 articles identified by database searches, 149 were finally selected for screening after removing duplicates. A general PRISMA flow chart was devised detailing the number of studies excluded at each stage of screening (Figure 1). Six additional PRISMA flow charts are provided; these detail the article screening and removal processes separately for each individual CPSS (Supplementary Material). An analysis of 21 full-text articles was performed in order to determine their eligibility for our review. These 21 articles fulfilled the inclusion criteria, so were subjected to the data extraction (Table 1) and quality assessment (Table 2) processes. They were all published between 2012 and 2020. While 5 studies were uncontrolled clinical trials [91,92,93,94,95], the remaining 16 did have one or more control group/s. Ten studies were performed in Europe (Sweden, United Kingdom, Spain, Denmark, Italy, Greece and Cyprus) [96,97,98,99,100], five in the USA [101,102,103,104,105], four in Iran [106,107,108,109], one in Canada [110], and one in Japan [111]. More details can be found in Table 1.

### 3.2. Participants

The 21 selected studies on ACT for CPSS [91,92,93,94,95,96,97,98,99,100,101,102,103,104,105,106,107,108,109,110,111] included 1090 individuals who completed the pre/post-test phases (average of 52 subjects per study; range: 7 to 141 participants). Approximately half of the participants were included in the ACT group (*n* = 601) and the others were included in control groups (*n* = 487). The mean age of the subjects was 42.88 years, and there was no significant age difference between participants in the ACT group and those in the control group in any study, with the exceptions of Aghalar et al. [106], in which the ACT group participants were significantly older than controls, and Kamalinejad et al. [107], in which participant ages were not directly reported. Regarding sex, the samples of seven studies were composed entirely of women [93,95,96,97,100,105,109], while in ten studies there was a clear female predominance (≥80%) [10,91,92,98,99,100,103,104,108,111]. Finally, four studies did not report information about subject sex [94,102,106,107].

### 3.3. Effectiveness of Acceptance and Commitment Therapy in the Treatment of Central Pain Sensitization Syndromes

No studies were found on the effectiveness of ACT in some CPSS, like CTTH, IC and TMD, indicating a need for further studies in this area. Regarding the effectiveness of ACT in IC patients, although no study was found on this topic, it seems that a similar psychological treatment, such as mindfulness, might be useful to reduce the symptomatology and improve some psychological aspects [112]. Therefore, further research to determine the effectiveness of different psychological approaches for IC patients is necessary, and seems justified based on the evidence regarding the relationship of this chronic pain condition with several psychosocial factors (anxiety, stress, trauma, depression, quality of life, etc.) [113]. Similarly, despite the relevance of this TMD, there are no articles about the effectiveness of ACT for TMD. In the same line, in spite of the importance of CTTH, there are no articles about the effect of ACT on CTTH. Our initial research revealed 28 articles, but after the selection and analysis process, none remained that fulfilled the requirements for inclusion in the review. The PRISMA flow chart is shown in the supplementary material. In addition, we found one study [109] on migraine that included patients with CTTH.

#### 3.3.1. Fibromyalgia Syndrome

Eight relevant articles were included in the review related to the effectiveness of ACT for FMS, although one of these did not exclusively recruit FMS patients; although the proportion of FMS patients exceeded 70%, subjects with other CPSS were also present in relatively high proportions (see Table 1 for details) [98].

Among the included studies on ACT for FMS, three found a decrease in pain [95,97,110], even though pain reduction is not the main objective of ACT therapy. Another study reported better reappraisal of pain, despite no change in pain thresholds or levels [96]. Another study observed an improvement only in pain-related functioning [100], while others reported no or little change in pain intensity [93,110]. Other clinical symptoms such as fatigue and sleep problems, did not improve after ACT [93,95,100].

However, there was greater agreement among the studies regarding the ability of ACT to improve anxiety and depression [93,95,96,97,100,110]. Other psychological factors, like pain acceptance [97,110], psychological flexibility [95,100] and self-efficacy [100], increased after ACT and could have mediated the clinical improvements seen in these patients. The clinical improvement was also supported by the self-perceptions of the FMS patients in some studies [96,97].

Regarding general wellness and social aspects, improvements in quality of life and disability [95,97,98,100,110], greater involvement in social activities [93] and better intimate relationships [105] were revealed by post-intervention evaluations.

Finally, ACT was superior to pharmacological [97,110] and psycho-education interventions [105], as well as WL conditions [95,96,100]; also, the effectiveness of ACT did not vary by intervention duration [98]. Therefore, ACT interventions seem to be useful for FMS, especially to treat the psychological, social and clinical symptoms that can impair quality of life and cause disability. Nevertheless, ACT also has utility to attenuate the pain experienced by these patients.

#### 3.3.2. Irritable Bowel Syndrome

Six relevant studies were included in this review regarding the effectiveness of ACT for IBS. ACT interventions promote more positive perceptions of the illness, in terms of acute pain/stress, by increasing acceptance of the disease and adherence to values [106]. Similarly, another study showed that ACT improved optimism and well-being [107], and an improvement of the psychological capital (self-perception of success and tolerance to problems) of IBS patients has also been observed [108]. Studies using a 1-day intervention or bibliotherapy program found that ACT reduced depressive mood [108,111]. However, these results have to be interpreted cautiously due to several limitations of the studies (see Risk of bias section and Table 2).

In general, there is controversy regarding the effectiveness [91,92], or ineffectiveness [111], of ACT in reducing the severity of IBS symptoms, and regarding its capacity to improve the quality of life of IBS patients, and change value-related behaviors [91,92]. A potential reason for ineffectiveness could be the lack of capacity of these programs to foster consistent daily practice. Even so, a 1-day ACT intervention can be useful to increase acceptance of IBS [91,92]. In general, ACT appears as a useful therapy for IBS patients, although further research on more complete ACT programs is still necessary. Better control of possible confounding variables (e.g., medication use or life events) is also required.

#### 3.3.3. Migraine

Seven relevant articles were included in the review regarding the effectiveness of ACT for migraine. ACT was shown to reduce the sensory and emotional dimensions of clinical pain in these patients [109], but not the sensory dimension [109]. A reduction in pain severity has also been reported [99]. Migraine-related symptoms, such as depression and disability, were also found to be decreased by ACT [103].

Regarding emotional variables, ACT seems to reduce affective distress [109], as well as levels of anxiety and depression [99,103,104]. Antidepressant intake did not moderate the effects of the treatment on depression severity at the 3-month follow-up [103]. Future research should take comorbid depression into account, given that depression is associated with poorer medical prognosis, decreased quality of life, and increased risk of disability and suicidality in patients with migraine [104].

With respect to quality of life, ACT has been shown to increase quality of life [99,103,104], levels of functioning [103], psychological well-being and the quality of social relationships [104], as well as to reduce disability [99,101,103,104]. Moreover, ACT reduces pain fusion and pain avoidance, and increases adherence to values [99].

Some studies also confirmed a significant reduction in the number of days of headache per month, and of medication intake per month, in their ACT groups [94,101]. Additionally, a pilot study on a 1-day ACT treatment reported that ACT plus Migraine Education (ACT-ED) led to significant improvements in headache frequency, headache severity, medication use, and headache-related disability, together with a reduction in the number of visits to healthcare professionals [101]. However, differences in headache outcomes between ACT-ED and treatment-as-usual (TAU) groups were not statistically significant over time (i.e., the treatment by time interaction was non-significant) [101]. These results complement those of a previous report showing higher efficacy of ACT-ED than TAU for treating depression and disability in migraine patients [103]. Dindo et al. [104] also reported that participants in their ACT-ED condition exhibited significantly greater improvements in depressive symptoms, general functioning, and migraine-related disability than patients in WL and TAU groups. Some years later, a randomized clinical trial of a 1-day ACT intervention showed improvements in depression, anxiety, headache-related disability, and quality of social relationships in depressed migraine patients [104]. No significant mediating effects of gender, race, education, income, or medication use were observed in the ACT-ED or Support plus Education (S-ED) groups [104].

ACT has been proposed as an adjunctive or alternative to pharmacologic therapies for the management of episodic migraine [102], both in the outpatient and hospital setting [109]. Despite previous positive evidence, further studies are required. The ACT approach, which focuses on acceptance and value-based activities, is a promising strategy to improve disability, functioning, and quality of life among patients with migraine Therefore, further research is needed to determine the conditions that best promote its effectiveness.

#### 3.3.4. Risk of Bias

The ROB evaluation was performed by two independent researchers (C.M.G.S. and P.d.C.). The initial agreement rate was 95%. Consensus was achieved either through discussing the interpretation of the criteria again, or via the involvement of a third independent reviewer (G.A.R.P). The ROB evaluation revealed that 12 studies were of low quality [93,94,95,98,101,102,103,105,106,107,108,109], 5 were of moderate quality [91,92,96,103,111], and 4 were high quality [97,99,100,110]. Details on the ROB assessments can be found in Table 2.

Other limitations were identified, such as imprecise specification of diagnostic criteria [93,95,102,105,107,108], lack of specificity regarding features of the ACT intervention [101,102,111], not enough patients for the differentiation of migraine with aura and migraine without aura [105,110], failure to report follow-up assessments [93,106,109], performance of only a 1-day ACT session [91,92,101,103,104,111], no indication of the sample sex ratio [94,102,106,107], failure to report analyses by sex [91,92,93,95,96,97,98,99,100,101,103,104,105,108,109,110,111], failure to specify the method used to determine the sample size [91,94,95,96,101,102,103,104,105,106,107,108,109,111], and failure to report any measure of the effect size [93,94,96,98,102,107].

## 4. Discussion

ACT seems to have efficacy for the treatment of the symptoms associated with chronic pain in CPSS, at least FMS, IBS and migraine. Nevertheless, there were no studies available for TTH, IC or TMD. Although ACT was not primarily intended to reduce pain, it may be able to reduce the subjective intensity thereof, improve adaptation to illness at least in FMS, IBS and migraine patients [91,92,94,95,97,101,102,110] and reduce medication intake in migraine patients [94,101,102]. However, ACT does not seem to change acute pain perception in CPSS patients, specifically in FMS and migraine patients [96,109], modify post fulfilment of diagnostic criteria in IBS patients [92] or improve symptoms such as fatigue or sleep disturbance in FMS [93,95,110]. Furthermore, while the effectiveness of ACT for reducing the severity of clinical symptoms has been demonstrated in some studies focused on IBS [91,92], others found no such effect in FMS and IBS patients [100,111]. In Haugmark [114] in a recent systematic review and meta-analyses showed small to moderate effects in favor of acceptance-based interventions in FMS patients compared to controls in pain, depression, anxiety, sleep quality and health-related quality of life [114].

There is good agreement regarding the benefits of ACT for reducing symptoms associated with pain, such as anxiety and depressive mood in FMS, IBS and migraine patients [93,95,96,97,99,100,103,104,108,109,110,111]. Psychological factors like pain acceptance in FMS and IBS patients [91,97,105], psychological flexibility in FMS and IBS patients [91,95,100], optimism about the illness in IBS patients [106,107], self-efficacy in FMS and IBS patients [100,108] and adherence to values in IBS patients [91,106] have been suggested to mediate the clinical improvements associated with ACT. In line with this, gender, race, education, income, and medication were discounted as possible moderators at least in migraine patients [103,104]. However, further investigation appears necessary, since there are controversies regarding certain of the proposed mediating variables, such as possible changes in avoidance behaviors over time in IBS patients [92] and insufficient persistence of pain acceptance in migraine [99].

Clear improvements in quality of life (mostly health-related quality of life) and general functioning have also been found in FMS, IBS and migraine patients, especially in terms of disability [91,95,97,98,99,100,101,102,104,107,109,110]. Some studies also observed an improvement in social functioning, specifically in FMS and migraine patients [93,104,105].

All of these outcomes should be further studied in future ACT clinical trials including CPSS patients. It seems that the ACT intervention has to be properly administered (i.e., adherence to the basic principles of the therapy) to obtain better outcomes compared to control conditions like WL, other treatments (such as those involving pharmacological agents) in FMS and migraine patients [97,103,110], and psychoeducational interventions in FMS [105]. However, it is not clear that extended ACT treatments are more advantageous than briefer options like 1-day or bibliotherapy interventions; studies have reported good results using short interventions in FMS, IBS and migraine patients [92,101,103,104,111], while a larger study found in no difference in effectiveness according to the duration of ACT interventions in FMS patients [100]. In general, applying ACT in CPSS patients seems to reduce clinical symptoms and improve their health-related quality of life, which could be a product of greater psychological flexibility in the way of greater pain acceptance and adherence to values, and lower cognitive fusion and experiential avoidance. Besides, these effects not only seem to be observed in the short-term, but also after a long period at follow-up for ACT interventions on FMS, IBS, and migraine patients (≥six months) [91,92,95,97,99,102,104,107].

A major limitation of this review is that most participants were female. However, as noted previously, the prevalence of CPSS is higher in women than men. Another limitation is the differences in age and measured variables among the reviewed studies. Additionally, the failure to report effect sizes by some studies limited the interpretation of the results. Moreover, the presence of psychiatric comorbidities and the non-control of other possible mediator variables (e.g., face-to-face vs. online intervention) in the reviewed studies were other limitations that should be taken into consideration. In this sense, depression usually accompanies chronic pain, being a common comorbid condition in chronic pain disorders [115], including chronic migraine [116,117]. Chronic pain, as a stress generator, is a critical factor for the development of depression, and their coexistence tends to further aggravate the severity of both disorders. Unfortunately, the nature of the pain-depression association remains unclear, which is a serious problem for the management of chronic pain-induced depression [115]. Besides, due to the complexity of this comorbidity, it is important to address both pain and depressive symptoms when evaluating treatment options [118]. Specifically, in migraine, the comorbidity of depression and migraine is a major health concern as it is related to a poorer prognosis and quality of life [101,103,104]. Previous authors have suggested a shared etiology or underlying pathway for depression and migraine [119,120], which seems to be bidirectional [121]. Based on the likely associations between presence of depression and increased migraine-related burden and risk of disease progression, it is crucial to understand the impact of migraine treatment on the manifestation and management of both pain and depression [122]. Potential treatments for chronic migraine seem also to reduce psychiatric comorbidities [122]. L. Dindo et al. [101] reported that 1-day ACT-ED workshop might be a promising approach to the treatment of depression and disability in migraine patients. In the same line, a 1-day ACT-ED workshop targeting psychological flexibility may produce benefits for patients with comorbid migraine and depression [104]. According to the need for studies that jointly analyze migraine pain and depression, we included the papers of L. Dindo in the current systematic review. However, based on the possible bias and limitations in controlling the effect of comorbid depression on the effectiveness of ACT, the studies of L. Dindo et al. [101,103,104] were included in the Risk of Bias section.

A strength of our study was that we strictly followed a systematic methodological approach in accordance with the study protocol, which was registered at PROSPERO prior to beginning recruitment and prepared in accordance with the PRISMA guidelines [90]. Moreover, the literature search involved several databases and the screening, selection and data extraction processes were performed by independent authors, thereby minimizing the risk of selection bias.

Related to the clinical relevance of our results, this systematic review revealed a marked lack of studies on the effectiveness of ACT for CPSS, especially in CTTH, IC and TMD (no published studies available). As we previously explained, these CPSS are common reasons for medical demands and have a high personal and socioeconomic impact. These negative impacts have been confirmed in IBS [123,124,125], FMS [126,127,128] and migraine patients [129,130]. Therefore, the effectiveness of ACT in these syndromes can contribute to reduce their negative consequences in both patients and relatives, as well as reduce the costs for the health system. This review also provides hypothesis and certain evidence about the mediating processes such as pain acceptance, cognitive defusión, values and mindfulness (in general psychological flexibility), responsible for reported success in pain management, which could help in the implementation and development of psychological intervention programs for these populations.

In closing, it is necessary to continue exploring the effectiveness of ACT therapy for CPSS, taking previous results into account. Further research in CPSS should differentiate between migraine patients with aura and those without aura, and in general, all studies should better control the possible psychiatric comorbidities, especially depression and anxiety. While the treatment of CPSS requires several types of socioeconomic and health resources, advances in ACT could benefit not only the patients, but also society as a whole due to its low cost and the possibility to be applied in different contexts, such as face-to-face or online formats. To sum up, ACT appears to have a positive effect on the symptomatology of CPSS, and also improves the quality of life related to health of these patients.

## Figures and Tables

**Table 1:** Characteristics of selected studies on the effectiveness of acceptance and commitment therapy for central pain sensitization syndromes.

Fibromyalgia Syndrome				
First Author (Publication Year), Study Name, Country	Study Design/ Diagnostic Technique	Sample Size, Age (Mean ± SD)	Period and Treatment Characteristics	Variables and Results
Jensen et al., 2012. Cognitive Behavioral Therapy increases pain-evoked activation of the prefrontal cortex in patients with fibromyalgia. Sweden.	Randomized controlled clinical trial with follow-up. A previous diagnosis of FMS by primary care physicians. according to the 1990 ACR diagnostic criteria.	N = 43 women with FMS. ACT group = 25 (44.50 ± 1.50). WL group = 18 (46.90 ± 1.10).	Twelve weekly ACT sessions in groups of six patients. Improvement of functioning and life satisfaction by increasing the participants’ ability to behave in accordance with their values in the presence of interference (pain and distress). Follow-up: 3-month assessment.	Primary Outcomes: Anxiety: STAI */** Depression: BDI */** Event-related potentials (ERP)-P50 Functional magnetic resonance imaging (fMRI) during pressure-evoked pain: - Insula * - Cerebellum * - Thalamus and caudate * - Hippocampus * Pain intensity: 0-100 visual analog scale *; pressure pain thresholds
Steiner et al., 2013. Values-based action in fibromyalgia: results from a randomized pilot of acceptance and commitment therapy. USA.	Randomized controlled trial (pilot study). A diagnosis of FMS by a physician.	N = 28 women with FMS. ACT group = 18 (47.82 ± 12.91). Psycho-education group = 10 (50.00 ± 13.62).	Eight weekly sessions of ACT intervention based on the manual “Living Beyond Your Pain: Using Acceptance and Commitment Therapy to Ease Chronic Pain”. Follow-up: 12-week assessment.	Secondary Outcomes: Values: CVPI: - (1) Family */** (d = 0.75/0.81) - (2) Intimate relationships */** (d = 0.64/0.53) - (3) Friends - (4) Health - (5) Work * (d = 0.64) - (6) Personal growth and learning
Wicksell et al., 2013. Acceptance and commitment therapy for fibromyalgia: A randomized controlled trial. Sweden.	Randomized controlled clinical trial with follow-up. A previous diagnosis of FMS by primary care physicians according to the 1990 ACR diagnostic criteria + a weekly self-reported average pain intensity of >40 on a visual analogue scale (0–100).	N = 40 women with FMS (45.10 ± 6.60). ACT group = 23. WL group = 17.	Twelve weekly 90-min group ACT sessions with six participants per group. ACT intervention was organized into four phases: (1) preparing for behavioral change. (2) shifting perspective. (3) values-oriented behavior activation. (4) acceptance and cognitive diffusion. Follow-up: 3-month assessment.	Primary Outcomes: Anxiety: STAI: */** - State anxiety */** (d = 0.51/0.55) - Trait anxiety */** (d = 0.73/0.74) Depression: BDI */**. (d = 0.44/0.64) Impact of FMS: FIQ */**: (d = 0.41/0.66) Quality of life: SF-36 */**: - Mental quality of life */** (d = 0.84/1.06) - Physical quality of life */** (d = 0.19/0,28) Pain disability: PDI */**. (d = 0.75/0.73) Pain intensity: 0-100 pain numeric rating scale */**. *(d = 0.38/0.82)* Secondary Outcomes: Psychological inflexibility: PIPS. */** (d = 1.06/0.72) Self-efficacy: SES */**. (d = 0.74/0.38)
Ljótsson et al., 2014. Internet-Delivered Acceptance and Values-Based Exposure Treatment for Fibromyalgia: A Pilot Study. Sweden.	Uncontrolled trial (pilot study) with follow-up. A diagnosis of FMS confirmed by a physician.	N = 41 women with FMS (52.00 ± 9.00).	Ten weekly online treatment included acceptance, mindfulness, work on life values, and systematic exposure to FMS symptoms and FMS-related situations + regular contact with an assigned online therapist. Follow-up: 6-month assessment.	Primary Outcomes: Anxiety: HADS */**. (d = 0.75/0.90) Depression: HADS */**. (d = 0.80/1.03) Pain disability: PDI. */** (d = 0.82/0.87) Fatigue: FSS. */** (d = 0.75/0.62) Impact of FMS: FIQ */**: - General */** (d = 0.71/0.96) - Pain */** (d = 0.62 / 1.22) Quality of life: SF-12 */**: - Mental quality of life */** (d = 0.63/0.86) - Physical quality of life */** (d = 0.85/0.68) Secondary Outcomes: Psychological Inflexibility: PIPS */**. (d = 1.56/2.01)
Luciano et al., 2014. Effectiveness of group acceptance and commitment therapy for fibromyalgia: A 6-month randomized controlled trial (EFFIGACT study). Spain.	Randomized controlled clinical trial with follow-up. Self-rated fulfillment of the ACR 1990 criteria for FMS at a screening visit to a primary health care center.	N = 156 women with FMS. Final sample = 136. ACT group = 51 ACT (48.88 ± 5.94) Final sample = 45. Pharmacologic group = 52 (47.77 ± 5.87) Final sample = 44. WL group = 53 WL (48.28 ± 5.71) Final sample = 47.	Eight weekly sessions with exercises based on ACT and mindfulness practice. Follow-up: 6-month assessment.	Primary Outcomes: Anxiety: HADS */**. (d = 0.36/0.39) Catastrophizing: PCS */**. (d = 0.76/0.69) Clinical pain: visual analog scale. */** (d = 0.62/0.47) Depression: HADS. */** (d = 0.43/0.37) Impact of FMS: FIQ */**. (d = 1.43/1.43) Quality of life: EQ-5D */**. (d = 0.85/0.66) Secondary Outcomes: Acceptance of chronic pain: CPAQ */**. (d = 1.05/1.01) Effect sizes for the comparisons between ACT and pharmacologic group (the differences between ACT and WL were even larger).
Pedersen et al., 2018. Acceptance and Commitment Group Therapy for patients with multiple functional somatic syndromes: a three-armed trial comparing ACT in a brief and extended version with enhanced care. Denmark. †	Randomized controlled clinical trial. Diagnosis made by a physician using the Bodily Distress Syndrome (BDS) checklist. The diagnosis was established by a medical doctor after a thorough physical and psychological Assessment, including the SCAN diagnostic interview.	N = 180 patients with CPSS. Final sample = 139: Sample of patients suffering from one or more central sensitization syndromes (>70% FMS; >50% tension headache; >35% IBS). Extended ACT group = 59 (38.80 ± 8.00) [80% women]. Final sample = 44. Brief ACT group = 61 (38.70 ± 8.60) [87% women]. Final sample = 49. Enhanced care group = 60 (40.10 ± 8.50) [87% women]. Final sample = 46.	Extended ACT: Nine weekly 3-h ACT sessions during a 3-month period led by two therapists. Treatment based on hexaflex model. Brief ACT: A workshop involving up to 15 patients providing information about illness and an introduction to ACT concepts through psycho-education, experiential exercises and group discussions. Enhanced care: A 1–1.5-h session/consultation for enhancing the patient’s understanding of their symptoms and diagnosis, and to optimize the treatment initiatives. Follow-up: 6-, 14- and 20-month assessments.	Clinical improvement: CGI * Disability: WHODAS 2.0. Distress: SCL-92: - Anxiety. - Depression. Illness worry: 7-item Whiteley checklist. Quality of life: SF-36: - Mental quality of life. - Physical quality of life.
Simister et al., 2018. Randomized Controlled Trial of Online Acceptance and Commitment Therapy for Fibromyalgia. Canada.	Randomized controlled clinical trial with follow-up. A diagnosis of FMS by a medical professional according to the 1990 ACR diagnostic criteria for FMS.	N = 67 FMS patients (39.70 ± 9.36) [95% women]. ACT + TAU group = 34. TAU group = 33.	2-month online ACT protocol on a virtual platform. Seven treatment modules including a written unit with 5-8 pages on metaphors, experiential exercises, and introductory and recurring vignettes describing typical FMS experiences, along with videos and experiential homework tasks. * TAU = analgesics and other treatments like physiotherapy or physical exercise. Follow-up: 3-month assessment.	Primary Outcomes: Aerobic capacity: 6-min walk test. Catastrophizing: PCS.Clinical pain: SFMPQ. */** (d = 0.84/0.11) Depression: CES-D */**. (d = 0.87/0.56) Impact of FMS: FIQ */**. (d = 1.26/1.59) Kinesiophobia: TSK-11 */**. (d = 0.95/0.64) Physical exercise tolerance: 1-min sit-to-stand test. Sleep: PSQI. (d = 0.79/0.53) Secondary Outcomes: Acceptance of chronic pain: CPAQ */**. (d = 0.84/0.80) Cognitive fusion: CFQ */**. (d = 0.51/0.55) Mindfulness: FFMQ. Valued living: VLQ */**. (d = 0.51/0.46)
Gómez-Pérez et al., 2020. Brief Acceptance and Commitment Therapy for Fibromyalgia: Feasibility and Effectiveness of a Replicated Single-Case Design. Spain.	Quasi-experimental, replicated single-case/small group design. A previous diagnosis of FMS by a rheumatologist.	N = 7 women with FMS. Group ACT intervention = 4 (59.75 ± 7.27). Individual ACT intervention = 3 (65.00 ± 2.65).	Five weekly ACT sessions (a brief treatment protocol created by the group LabPsiTec): -1-h session of individual therapy. -1.5-h group session. Follow-up: None.	Primary Outcomes: Pain monitoring app: - Interference with sleep - Social activities - Fatigue - Sadness - Pain intensity
**Irritable Bowel Syndrome**				
Gillanders et al., 2017. An implementation trial of ACT-based bibliotherapy for irritable bowel syndrome. United Kingdom.	Uncontrolled pre/post-test study. Participants were diagnosed using the ROME III criteria for IBS by a consultant gastroenterologist.	N = 24 IBS patients (49.30 ± 14.90) [women = 19]. Final sample = 21.	A self-help book, “Better Living with IBS” (Ferreira & Gillanders, 2012), and the accompanying audio exercises on CD Follow-up: 2- and 6-month assessments.	Avoidance behaviors: BRQ. Diagnostic criteria for IBS: ROME III criteria **. (not estimated effect size) Quality of life: SF36. Symptom severity: SSS */** (ηp2 = 0.09/d = 0.33) Pain acceptance: AAQ-II: * (ηp2 = 0.08) - Activity engagement. - Willingness * (ηp2 = 0.14) Visceral sensitivity: VSI *. (ηp2 = 0.07)
Ferreira et al., 2018. Pilot study of acceptance and commitment therapy for irritable bowel syndrome: A preliminary analysis of treatment outcomes and processes of change. United Kingdom.	Uncontrolled pre/post-test study. Participants were diagnosed using the ROME III criteria for IBS by a consultant gastroenterologist.	N = 56 IBS patients (47.60 ± 13.00) [women = 52]. Final sample = 40.	One-day workshop about IBS (6 h) + a self-help book, “Better Living with IBS” (Ferreira & Gillanders, 2012), and the accompanying exercises in audio format. Follow-up: 6-month assessment.	Avoidance behaviors: BRQ */**. (d = 0.32/0.39) Quality of life: SF36 */** (d = 0.41/0.55) Symptoms severity: SSS */** (d = 0.41/0.47) Pain acceptance: AAQ-II */** (d = 0.32/0.50) Visceral sensitivity: VSI */**. (d = 0.76/1.10)
Kamalinejad et al., 2019. The Efficacy of Acceptance and Commitment Therapy on Psychological Well-Being and Optimism of Patients with Irritable Bowel Syndrome. Iran.	Controlled pre/post-test study. IBS patients referred from health centers in Tehran (Iran).	N= 60 IBS patients. ACT group = 30. Non-intervention group = 30.	Nine 90-min sessions of ACT therapy aimed at improving psychological well-being and optimism. Control group received no intervention. Follow-up: one assessment, timing not specified.	Optimism: LOT */**. Pessimism: LOT */**. Psychological well-being: RSPWB.*/** - Positive relationships */** - Autonomy */** - Environmental domination */** - Personal growth */** - Purposefulness in life */** - Admission */**
Mirsharifa et al., 2019. The Efficacy of Acceptance and Commitment Therapy (ACT) Matrix on Depression and Psychological Capital of the Patients with Irritable Bowel Syndrome. Iran.	Controlled pre/post-test study. Diagnosis of irritable bowel syndrome by a gastroenterology specialist.	N = 30 IBS patients between 19–60 years old (31.93) [19 women]. ACT group = 15. Non-intervention group = 15.	Six weekly 90-min ACT sessions based on the six principles of psychological flexibility. Two main elements comprised the intervention: (1) Reality vs. mental experience. (2) Behavior in line with values vs. behavior for escaping worries. Follow-up: none.	Depression: BDI *. (ηp2 = 0.08) Psychological capital (hope, efficacy, resilience and optimism): PCQ *. (ηp2 = 0.29)
Ito et al., 2020. Effectiveness of acceptance and commitment therapy for irritable bowel syndrome non-patients: A pilot randomized waiting list controlled trial. Japan.	Pilot randomized controlled trial. Japanese version of the IBS Severity Index (IBSSI).	N = 17 IBS patients [11 women]. ACT group = 9 (19.89 ± 1.36) [6 women]. WL group = 8 (19.63 ± 0.92) [5 women].	One-day ACT program consisting of a group session and 2-month self-help program + online value adherence quizzes. Follow-up: 2-month assessment.	Anxiety: STAI. Depression: BDI **. (d = 1.10) Cognitive fusion: CFQ. Mindfulness: FFMQ. Quality of life: SF-36; IBS-QOL. Pain acceptance and related actions: AAQ-II. Symptom severity: SSS
Aghalar et al., 2020. The Effectiveness of Acceptance and Commitment-Based Therapy on Perception of Disease in Patients with Irritable Bowel Syndrome. Iran.	Controlled pre/post-test study. The Rome III criteria (2006) for irritable bowel syndrome were used by a gastroenterology specialist for the diagnosis.	N = 30 IBS patients. ACT group= 15 (44.80 ± 4.72). Non-intervention group = 15 (33.43 ± 7.66).	Eight weekly 90-min ACT sessions based on an adaptation of the Zatel treatment protocol. Each session had different goals, techniques, and practices. At the end of each session, patients were required to practice at home, and progress was checked at the beginning of the next session. Follow-up: none.	Illness perception: BIPQ *: - Illness sequences * (d = 0.46) - Illness duration - Personal control * (d = 0.43) - Nature of illness * (d = 0.43) - Control through treatment * (d = 0.44) - Worrying about illness * (d = 0.40) - Knowing about illness - Affective response to illness * (d = 0.63)
**Migraine**				
Mo’tamedi et al., 2012. The effectiveness of a group-based acceptance and commitment additive therapy on rehabilitation of women outpatients with chronic headache: preliminary findings reducing 3 dimensions of headache impact. Iran.	Randomized pre/post-test control group design. ICDH, 2nd edition (2004).	N= 30 women outpatients diagnosed with primary chronic (migraine and tension-type) headache. ACT group = 15 (34.18 ± 7.39). - 8 chronic tension-type patients. - 7 chronic migraines (without aura) patients. Final sample = 11. TAU group = 15 (37.87 ± 8.74): - 10 chronic tension-type patients. - 5 chronic migraine (without aura) patients.	The ACT group, in addition to TAU, completed eight weekly sessions over 2 months. On average, each session lasted 90 min. A brief ACT orientation session was completed by each participant in the ACT group. The baseline and outcome assessment periods were 2 days in duration. The TAU group was given the opportunity to speak with the therapist and other participants about the problems they experienced with medication use, as well as other psychological problems. The therapist also provided the participants with guidance on problem-solving. Follow-up: none.	Primary Outcomes: Anxiety: STAI-Trait *. (d = 2.54) Clinical pain: Short-form McGill pain questionnaire: - Sensory * (d = 0.28) - Evaluative - Miscellaneous - Affective * (d = 1.35) - Total Migraine disability: MIDAS *. (d = 0.93)
Dindo et al., 2012. One-day behavioral treatment for patients with comorbid depression and migraine: a pilot study. USA.	Pilot study. A diagnosis of migraine made by a physician.	N= 45 patients with comorbid depression and migraine. ACT-ED group = 31 (32.50 ± 13.30) [97% women). WL/TAU group = 14 (33.50 ± 12.90) [86% women].	The ACT-ED group completed a 5-h workshop based on ACT and migraine education. Each ACT-ED workshop involved 5-8 patients and emphasized three topics. The education component (1 h) provided education about migraine. The ACT component (4 h) included training on acceptance and behavioral change. The WL/TAU group waited at least 12 weeks for treatment. Although no treatment was provided by the investigators during this time, the participants in the WL/TAU group completed the same clinical assessments as the ACT-ED group. Follow-up: 2, 6, and 12 weeks after the workshop.	Primary Outcomes: Depression: HRSD ** (d = 1.18); major depressive disorder criteria of SCID-IV **; IDAS ** (d = 0.87). Disability: WHO-DAS **. (d = 0.98) Headache disability: HDI **. (d = 1.03) Health related quality of life: SF-36 **. (d = 0.69) ** Significant results at the 12-weeks follow-up.
Dindo et al., 2014. One-day behavioral intervention in depressed migraine patients: effects on headache. USA.	Pilot study. Treatment trial. A diagnosis of migraine made by a physician.	N= 60 patients with comorbid migraine and depression. ACT-ED group = 38 (32.50 ± 12.60) [95% women] TAU group = 22 (29.60 ± 11.70) [91% women].	The ACT-ED group completed a 5-h workshop based on ACT and migraine education. Each ACT-ED workshop involved 5–8 patients. The migraine education component (1 h) provided education about migraine. Patients in the TAU group completed the same clinical assessments as the ACT-ED group. Follow-up: 3 months.	Primary Outcomes: Daily headache: headache frequency **/severity **, medication use**, disability **, and visits to a healthcare professional **.
Grazzi et al., 2019. ACT for migraine: effect of acceptance and commitment therapy (ACT) for high-frequency episodic migraine without aura: preliminary data of a phase-II, multicentric, randomized, open-label study. Italy.	Multicentre, phase-II, open, randomized trial. Diagnosis of high-frequency migraine without aura. ICHD-3 beta, 3rd edition (2013).	N= 50 patients with high-frequency episodic migraine without aura (18-65 years old). Still ongoing (24 patients).	Patients were randomized to one of the following treatment arms: Condition A: education of patients, followed by pharmacological prophylaxis for migraine. Condition B: education of patients, followed by pharmacological prophylaxis for migraine plus ACT. The ACT protocol consisted of 6 90-m weekly sessions and two supplementary sessions separated by a 15-day interval, wherein patients were trained in mindfulness and pain management. Patients were trained in small groups (5/8) by a therapist, and were instructed to practice at home for at least 10 min per day. Reported follow-up: 3 months.	Primary Outcomes: Daily headache diary: decrease in days of headache/month ** and medication intake/month **. Not reported Outcomes: Anxiety: HADS. Depression: HADS. Headache impact: HIT6. Migraine disability: MIDAS. Pain catastrophizing: PCS.
Dindo et al., 2020. One-Day Acceptance and Commitment Therapy Compared to Support for Depressed Migraine Patients: a Randomized Clinical Trial. USA.	Randomized clinical trial. ID Migraine: Brief self-administered migraine screening test (Lipton et al., 2003). A medical chart diagnosis of migraine.	N = 103 patients with comorbid depression and migraine. ACT-ED group = 56 (36.90 ± 14.90) [47 women] S-ED group = 47 (34.40 ± 12.60) [38 women].	ACT-ED. Two 1-day (5- to 6-h) interventions. The ACT-ED workshops lasted 5–6 h, involved 4–8 patients, and provided training in ACT and education about migraine. The S-ED workshop also lasted about 5–6 h and involved 4–8 patients. The same educational topics listed above about migraine were covered. Follow-up: 3 and 6 months after treatment.	Primary Outcomes: Anxiety: SIGH-A **. (d = 0.74) Depression: HRSD **. (d = 0.46) Disability: WHO-DAS**. (d = 0.23) Headache disability: HDI **. (d = 0.48) Quality of life: WHO-QOL **: - Psychological well-being ** (d = 0.44) - Social relationships - Environment - Physical health ** (d = 0.24) ** Significant results at the 6-month follow-up.
Vasiliou et al., 2020. Acceptance and Commitment Therapy for Primary Headache Sufferers: A Randomized Controlled Trial of Efficacy. Cyprus and Greece.	Randomized clinical trial. ICHD-3 beta, 3rd edition (2013) and a psychological evaluation by a doctoral-level clinical psychology trainee.	N = 94 patients with an 87.35% migraine diagnosis rate (43.00 ± 10.35) [84% women]. ACT group = 47 (42.89 ± 10.27) [74.53% women]. Final sample = 31. WL group = 47 (44.92 ± 10.43) [92.58% women]. Final sample = 30.	The authors developed an updated ACT process-based treatment guide consisting of three components: a therapist’s manual, a participants’ workbook, and 2 CDs. The 8 weekly, 1.5-h treatment sessions were conducted in groups of approximately 8 to 10 participants and two co-therapists. There was also an additional, final last session wherein participants were accompanied by their significant others. Follow-up: 3-, 6- and 12-month assessments (the final two were only for the ACT group).	Primary Outcomes: Anxiety: HADS. Clinical pain: GBPI: - Pain severity ** (RCI = 47%) Cognitive screening: MMSE. Depression: HADS. Headache disability: HDI-Func ** (RCI = 48%); HDI-Em ** (RCI = 33%). Medical utilization. Migraine-specific quality of life: MSQ v 2.1 **: - Role restrictive ** (RCI = 42%) - Emotional role ** (RCI = 32%) - Role preventive ** (RCI = 23%) ** Significant results at the 12-month follow-up. Secondary Outcomes: Chronic pain acceptance: Greek CPAQ **. (ηp2 = 0.14) Cognitive affective mindfulness: CAMS. Committed action: CAQ. Psychological inflexibility: Greek PIPS-II **: - Pain fusion ** (ηp2 = 0.13) - Pain avoidance ** (ηp2 = 0.12) Values: VQ: - Value progress - Barriers to value adherence ** (ηp2 = 0.04) ** Significant results at the 3-month follow-up in relation to post-test outcomes.
Grazzi et al., 2020. Acceptance and Commitment Therapy (ACT) vs Erenumab for High-Frequency Episodic Migraine Without Aura: Time to Take the Gloves Off! USA.	Longitudinal study. ICDH.	N= 40 patients with HFEM without aura. ACT group: 13 (42.10 ± 11.60). TAU group: 11 (41.80 ± 11.10). Erenumab group: 16 (45.70 ± 9.70).	The ACT protocol consisted of six 90-min small group sessions (once per week for 6 weeks) followed by two “booster” sessions. Training sessions involved structured behavioral education, experiential exercises and home assignments. The erenumab group was treated with 70 mg erenumab (per month as an adjunct to pharmacological prophylaxis. This group was not included in the original ACT project, and served as a further comparison group. Follow-up: 3- and 6-month assessments.	Primary Outcomes: Monthly migraine days **. Monthly medication intake **. Significant differences between ACT and TAU group (regarding the comparisons between ACT and Erenumab, there were hardly any differences between them). ** Significant results at the 6-month follow-up.

Note: * significant pre/post change (short-term effects). ** significant change at the last follow-up (long-term effects). *d* = Cohen’s d; *η_p_^2^* = partial squared eta; RCI = Reliable Change Index in percentage (available statistics for effect size). † Sample composed of patients suffering from one or more CPSS (FMS > 70%; Tension headache > 50%; and/or IBS >35%). Abbreviations: AAQ-II = Acceptance and Action Questionnaire; ACT: acceptance and commitment therapy; ACT-ED = Acceptance and Commitment Therapy plus Migraine Education; BDI = Beck Depression Inventory; BIPQ = Brief Illness Perception Questionnaire; BPI = Brief Pain Inventory; BRQ = IBS Behavioral Response Questionnaire; CAMS = Cognitive Affective Mindfulness Scale-Revised; CAQ = Committed Action Questionnaire; CES-D = Centre for Epidemiological Studies Depression Scale; CFQ = Cognitive Fusion Questionnaire; CGI = Clinical Global Improvement; CPAQ = Chronic Pain Acceptance Questionnaire; CPSS = Chronic Pain Sensitization Syndrome; CVPI = Chronic Pain Values Inventory; EQ-5D = Visual analogue scale of EuroQol; FFMQ = Five Facet Mindfulness Questionnaire; FIQ = Fibromyalgia Impact Questionnaire; FMS = Fibromyalgia Syndrome; FSS = Fatigue Severity Scale; GBPI: Greek Brief Pain Inventory; HADS = Hospital Anxiety and Depression Scale; HDI = Headache Disability Inventory; HDI-Func = Headache Disability Inventory-Functional; HDI-Em = Headache Disability Inventory-Emotional; HFEM = high-frequency migraine without aura; HIT6 = Headache Impact Test; HRSD = Hamilton Rating Scale for Depression; IBS = Irritable Bowel Syndrome; ICHD = International Classification of Headache Disorders; ICHD-3 beta = International Classification of Headache Disorders, third edition (beta version); IDAS = Inventory of Depression and Anxiety Symptoms; ID Migraine = Brief self-administered migraine screener; LOT = Life Orientation Test; MIDAS = Migraine disability assessment scale; MMSE = Mini-Mental Status Examination; MSQ v 2.1 = Migraine-Specific Quality of Life Questionnaire version 2.1; PCQ = Psychological Capital Questionnaire; PCS = Pain Catastrophizing Scale; PDI = Pain Disability Index; PIPS = Psychological Inflexibility in Pain Scale; PSQI = Pittsburgh Sleep Quality Index; QOL = Quality of Life Questionnaire; RSPWB = Ryff Scales of Psychological Well-Being; SCID-IV = Structured Clinical Interview for DSM-IV; SCL-92 = 92-item version of the Hopkins Symptom Checklist; SD = standard deviation; S-ED = support plus migraine education; SES = Self-Efficacy Scale; SF-12 = Short Form-12 Health Survey; SF-36 = Short Form-36 Health Survey; SFMPQ = McGill Pain Questionnaire – short form; SIGH-A = Structured Interview Guide for the Hamilton Anxiety Rating Scale.; SRS V.3.0 = Session Rating Scale-version 3; SSS = Symptom Severity Scale; STAI = Spielberger Trait-State Anxiety Inventory; TAU = treatment-as-usual; TSK-11 = Tampa Scale for Kinesiophobia-11; USA = United States of America; VLQ = Valued Living Questionnaire; VQ = Valuing Questionnaire; VSI = Visceral Sensitivity Index; WHO = World Health Organization; WHO-QOL: World Health Organization Quality of Life; WHODAS 2.0 = World Health Organization’s Disability Assessment Schedule, Version 2.0; WL = waiting list.

**Table 2:** Risk of Bias Assessment of relevant eligible studies.

First Author (Year)	Random Sequence Generation (Selection Bias)	Allocation Concealment (Selection Bias)	Blinding of Participants and Personnel (Performance Bias)	Blinding of Outcome Assessment (Detection Bias)	Incomplete Outcome Data (Attrition Bias)	Selective Reporting (Reporting Bias)	Other Bias	General Assessment (Low, Medium, High)
Fibromyalgia Syndrome								
Jensen et al., 2012.	L	L	M	L	M	L	Yes	Medium
Steiner et al., 2013.	M	L	H	H	H	H	Yes	Low
Wicksell et al., 2013.	L	L	L	L	M	L	Yes	High
Ljóntsson et al., 2014.	M	H	M	H	L	L	Yes	Low
Luciano et al., 2014.	L	L	M	L	L	L	Yes	High
Pedersen et al., 2018. †	M	L	H	H	H	H	Yes	Low
Simister et al., 2018.	L	L	L	L	L	L	Yes	High
Gómez-Pérez et al., 2020.	H	L	H	H	H	M	Yes	Low
Irritable Bowel Syndrome								
Gillanders et al., 2017.	M	L	H	H	M	L	Yes	Moderate
Ferreira et al., 2018.	M	L	H	H	M	L	Yes	Moderate
Kamali-Nedjad et al., 2019.	H	M	H	H	H	H	Yes	Low
Mirsharifa et al., 2019.	H	L	H	H	H	H	Yes	Low
Aghalar et al., 2020.	H	L	H	H	M	H	Yes	Low
Ito et al., 2020.	M	L	H	H	M	L	Yes	Moderate
Migraine								
Mo’tamedi et al., 2012.	L	H	H	H	L	L	Yes	Low
Dindo et al., 2012.	H	H	H	H	L	L	Yes	Low
Dindo et al., 2014.	H	H	H	H	L	L	Yes	Low
Grazzi et al., 2019.	L	H	H	H	H	H	Yes	Low
Dindo et al., 2020.	L	L	L	H	L	L	Yes	Moderate
Vasiliou et al., 2020.	L	L	L	L	L	L	No	High
Grazzi et al., 2020.	L	H	H	H	H	H	Yes	Low

Note: L: Low, M: Medium, H: High. † Sample composed of patients suffering from one or more CPSS (FMS > 70%; Tension headache > 50%; and/or IBS > 35%).

## Data Availability

The datasets generated and/or analysed during the current study are available from the corresponding author.

## Supplementary Materials

The following are available online at https://www.mdpi.com/article/10.3390/jcm10122706/s1. Supplementary material associated with this article can be found in the online version.

## Abbreviations

ACT	Acceptance and Commitment Therapy.
CNS	Central Nervous System
CPSS	Central Pain Sensitization Syndrome.
CTTH	Chronic tension-type headache.
FMS	Fibromyalgia Syndrome.
HRQoL	Health Related Quality of Life.
IBS	Irritable Bowel Syndrome.
IC	Interstitial Cystitis.
ICHD-3 beta	International Classification of Headache Disorders, third edition (beta version).
TMD	Temporomandibular disorder.
TTH	Tension-Type Headache.
USA	United States of America.
