# Supplementary material for: Effectiveness of Acceptance and Commitment Therapy in Central Pain Sensitization Syndromes: A Systematic Review
Source: J Clin Med. 2021 Jun 19;10(12):2706. doi: 10.3390/jcm10122706 (PMC8235706; doi:10.3390/jcm10122706)

PRISMA 2009 Flow Diagram _ Fibromyalgia Syndrome

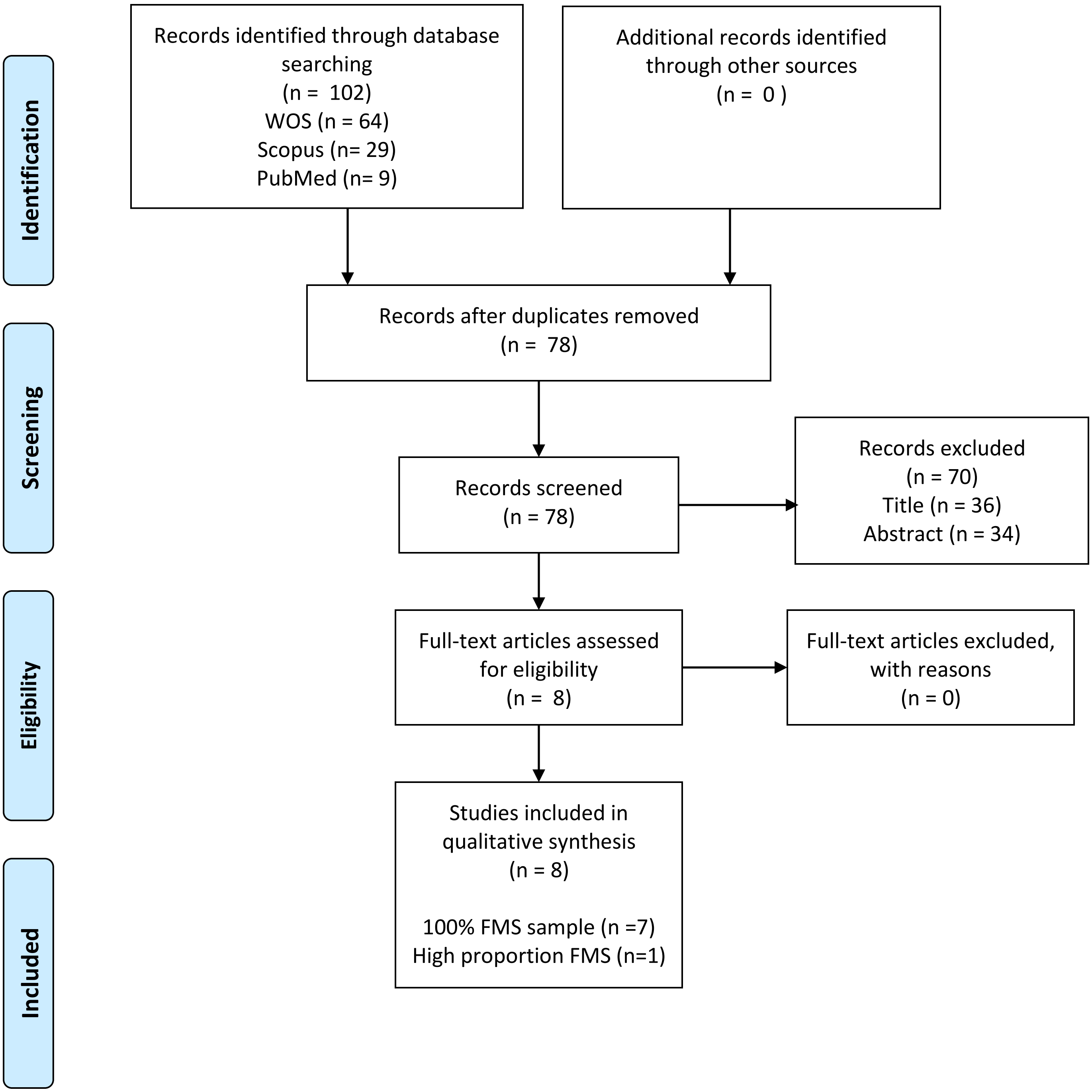

Note: FMS = Fibromyalgia Syndrome.

PRISMA 2009 Flow Diagram _ Irritable Bowel Syndrome

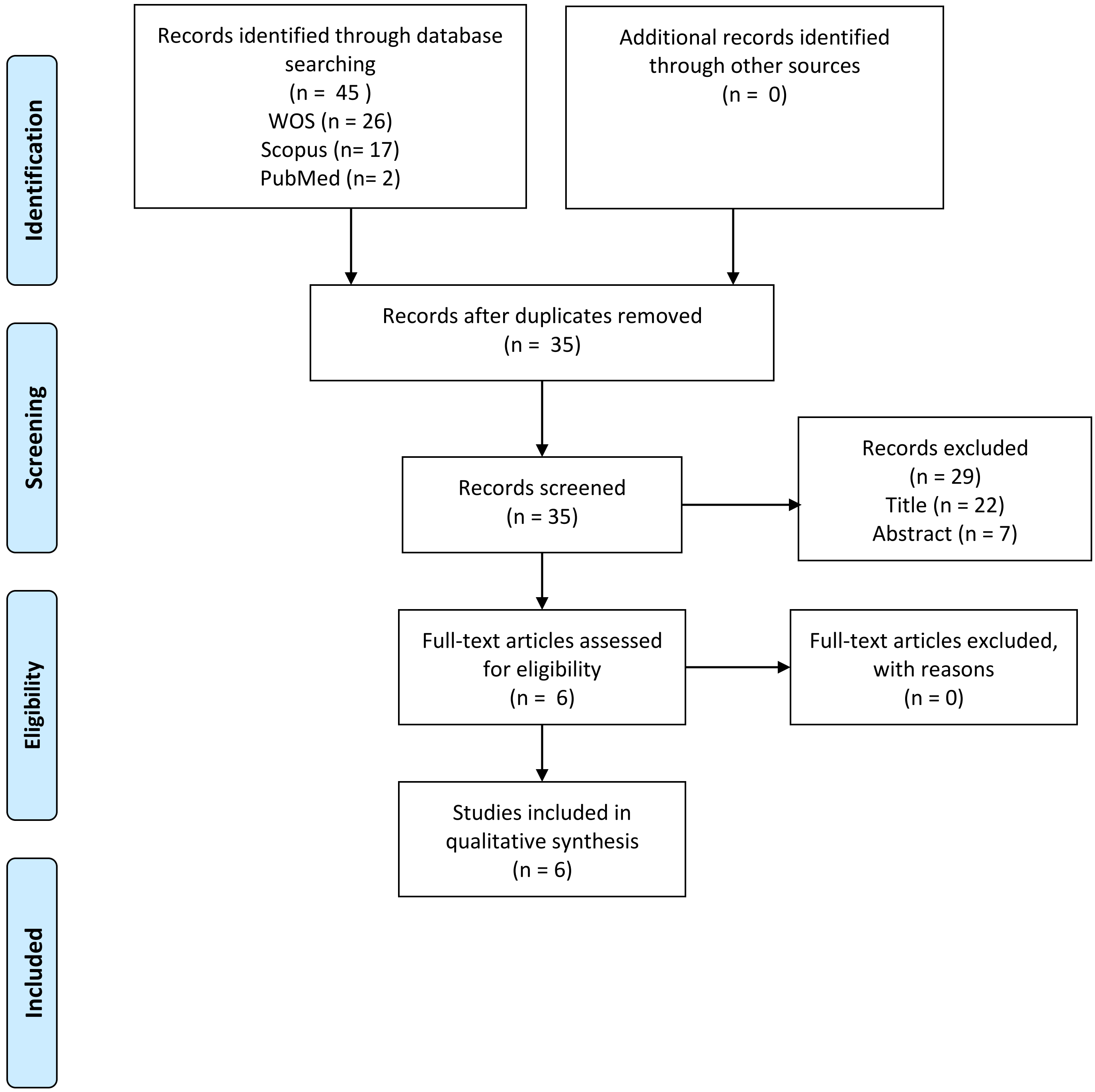

PRISMA 2009 Flow Diagram _ Intersticial Cystitis

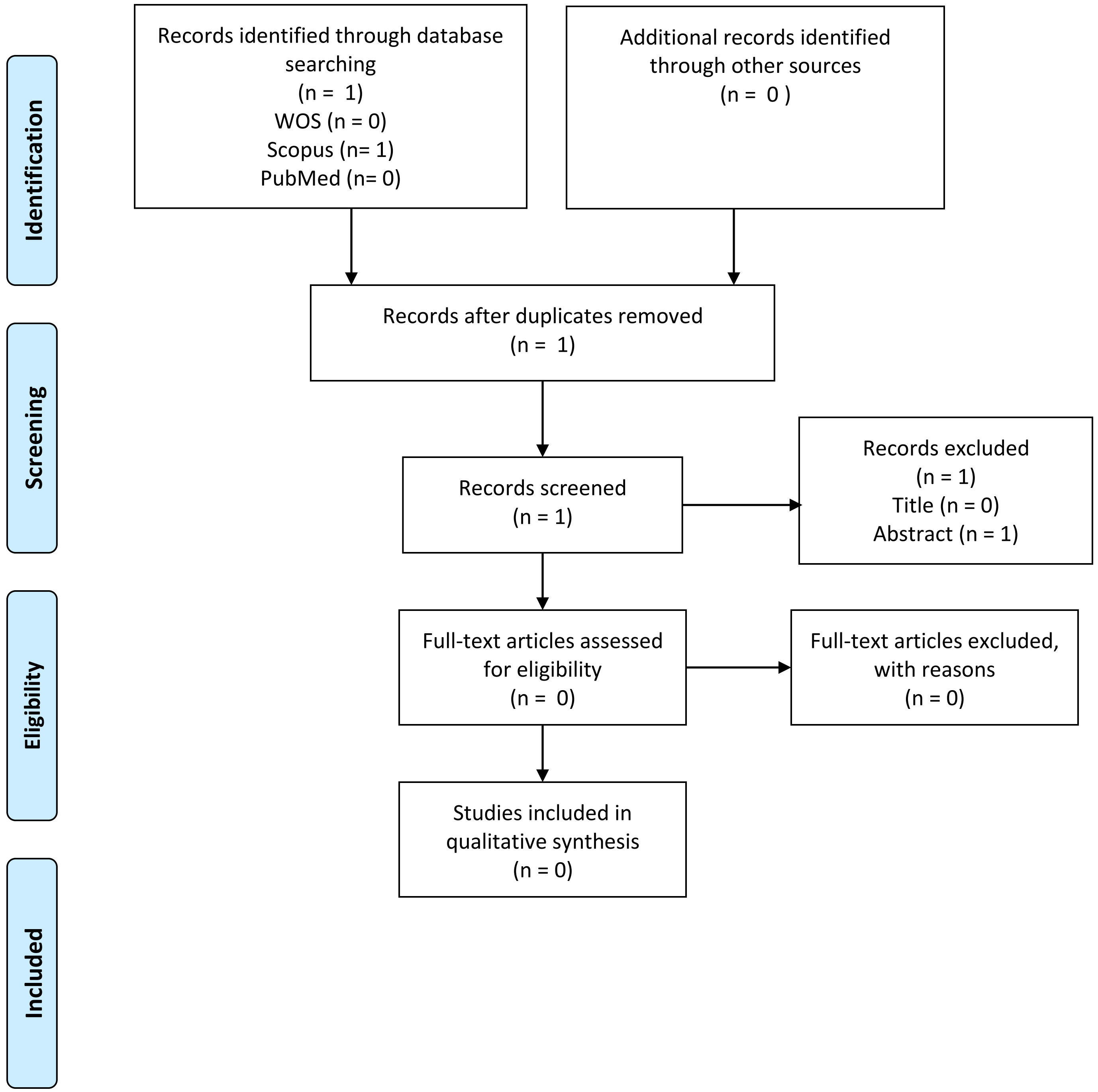

PRISMA 2009 Flow Diagram _ Temporomandibular Disorder

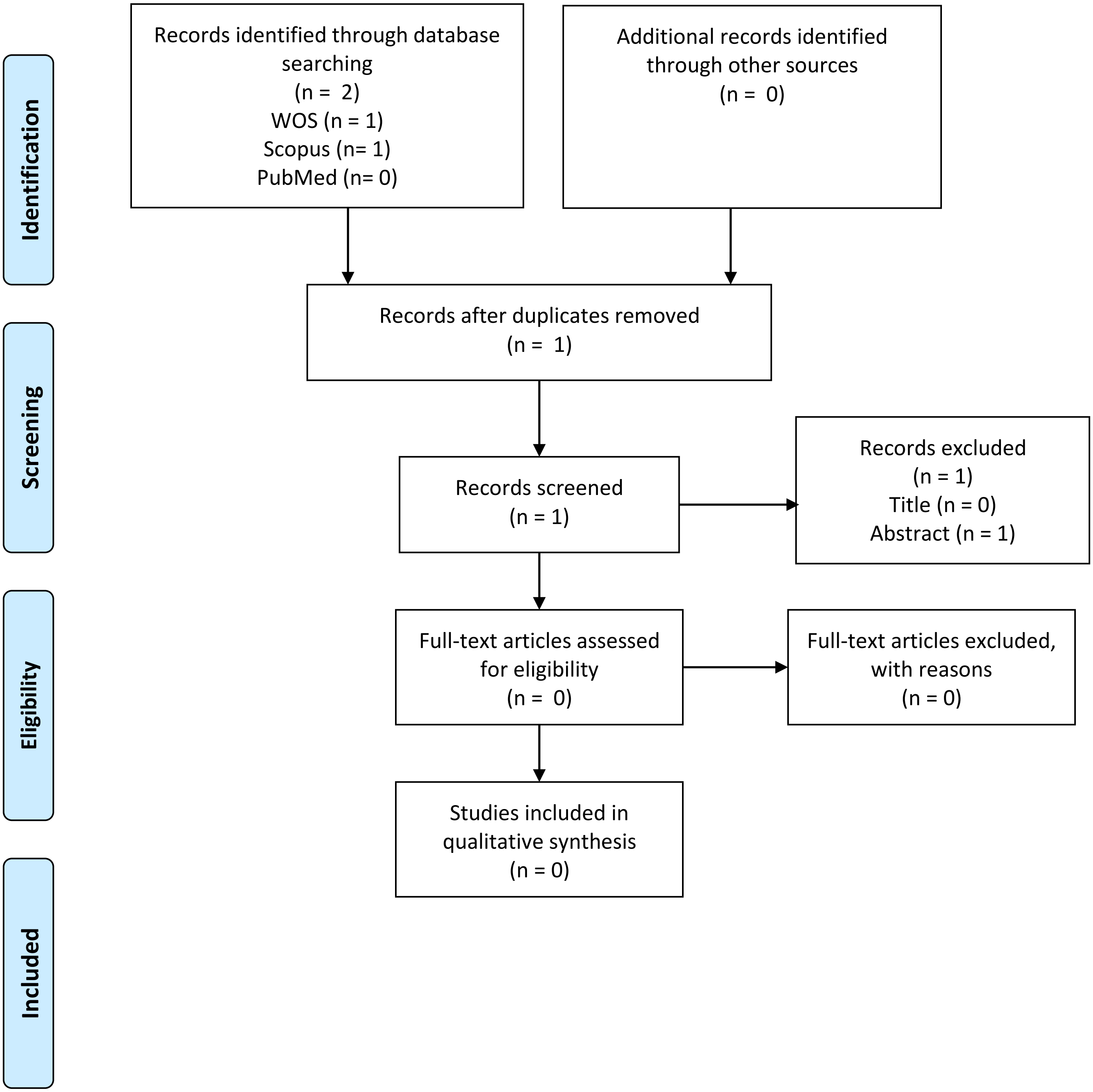

PRISMA 2009 Flow Diagram _ Chronic tension-type headache

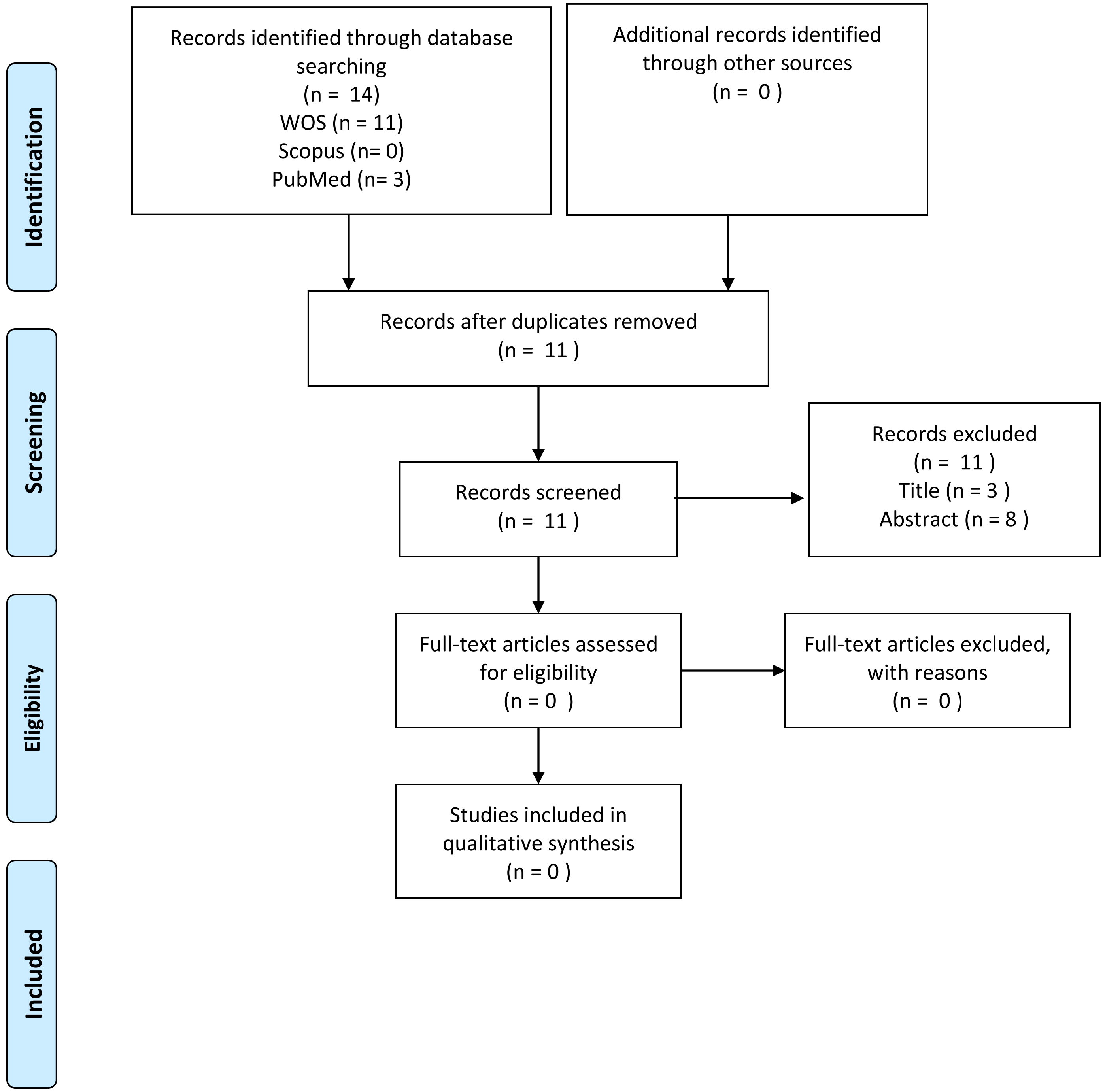

PRISMA 2009 Flow Diagram _ Migraine

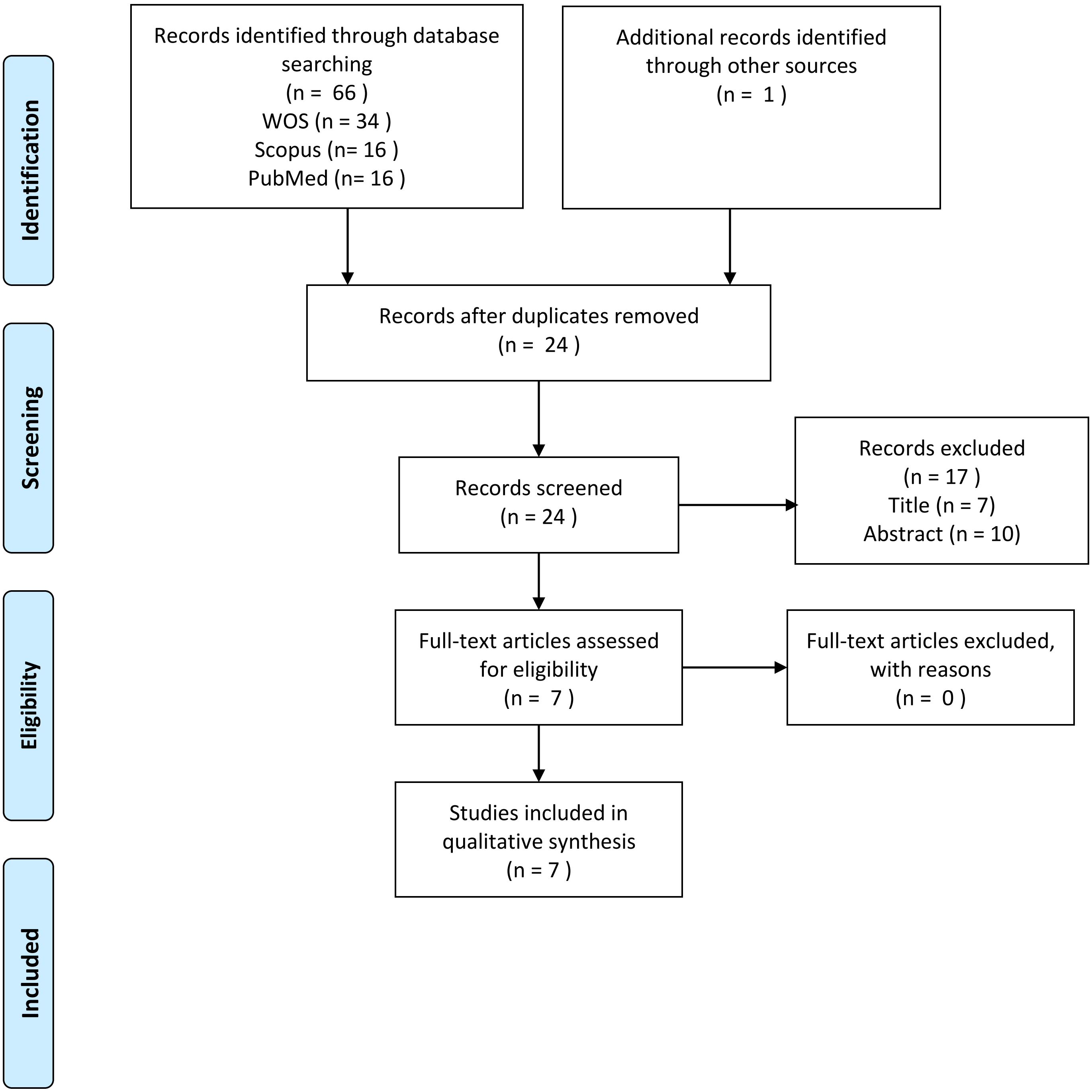

Supplement: Supplementary file 1 [file jcm-10-02706-s001.zip › jcm-1163306-supplementary.pdf]
